# Venezuelan Equine Encephalitis Virus Induces Apoptosis through the Unfolded Protein Response Activation of EGR1

**DOI:** 10.1128/JVI.02827-15

**Published:** 2016-03-11

**Authors:** Alan Baer, Lindsay Lundberg, Danielle Swales, Nicole Waybright, Chelsea Pinkham, Jonathan D. Dinman, Jonathan L. Jacobs, Kylene Kehn-Hall

**Affiliations:** a National Center for Biodefense and Infectious Diseases, School of Systems Biology, George Mason University, Manassas, Virginia, USA; b MRIGlobal, Global Health Security, Rockville, Maryland, USA; c University of Maryland, Department of Cell Biology and Molecular Genetics, College Park, Maryland, USA

## Abstract

Venezuelan equine encephalitis virus (VEEV) is a previously weaponized arthropod-borne virus responsible for causing acute and fatal encephalitis in animal and human hosts. The increased circulation and spread in the Americas of VEEV and other encephalitic arboviruses, such as eastern equine encephalitis virus and West Nile virus, underscore the need for research aimed at characterizing the pathogenesis of viral encephalomyelitis for the development of novel medical countermeasures. The host-pathogen dynamics of VEEV Trinidad donkey-infected human astrocytoma U87MG cells were determined by carrying out RNA sequencing (RNA-Seq) of poly(A) and mRNAs. To identify the critical alterations that take place in the host transcriptome following VEEV infection, samples were collected at 4, 8, and 16 h postinfection and RNA-Seq data were acquired using an Ion Torrent PGM platform. Differential expression of interferon response, stress response factors, and components of the unfolded protein response (UPR) was observed. The protein kinase RNA-like endoplasmic reticulum kinase (PERK) arm of the UPR was activated, as the expression of both activating transcription factor 4 (ATF4) and CHOP (DDIT3), critical regulators of the pathway, was altered after infection. Expression of the transcription factor early growth response 1 (EGR1) was induced in a PERK-dependent manner. EGR1^−/−^ mouse embryonic fibroblasts (MEFs) demonstrated lower susceptibility to VEEV-induced cell death than isogenic wild-type MEFs, indicating that EGR1 modulates proapoptotic pathways following VEEV infection. The influence of EGR1 is of great importance, as neuronal damage can lead to long-term sequelae in individuals who have survived VEEV infection.

**IMPORTANCE** Alphaviruses represent a group of clinically relevant viruses transmitted by mosquitoes to humans. In severe cases, viral spread targets neuronal tissue, resulting in significant and life-threatening inflammation dependent on a combination of virus-host interactions. Currently there are no therapeutics for infections cause by encephalitic alphaviruses due to an incomplete understanding of their molecular pathogenesis. Venezuelan equine encephalitis virus (VEEV) is an alphavirus that is prevalent in the Americas and that is capable of infecting horses and humans. Here we utilized next-generation RNA sequencing to identify differential alterations in VEEV-infected astrocytes. Our results indicated that the abundance of transcripts associated with the interferon and the unfolded protein response pathways was altered following infection and demonstrated that early growth response 1 (EGR1) contributed to VEEV-induced cell death.

## INTRODUCTION

Venezuelan equine encephalitis virus (VEEV) is a New World alphavirus in the family Togaviridae that is endemic to the Americas. VEEV is a positive-strand RNA virus that is transmitted by mosquitoes and that is naturally present in rodent reservoirs (1). There are six subtypes that are categorized by their geographic range and pathology in equines and humans. The two epizootic strains, IA/B and IC, arose from mutations among the enzootic strains (2). The IA/B and IC strains are of particular concern due to increased rates of morbidity and mortality and the risks associated with viral amplification and potential species spillover (2). In humans, VEEV causes a febrile illness typified by fever, malaise, and vomiting. In some cases, infection progresses to the central nervous system (CNS) and neurological symptoms, such as confusion, ataxia, and seizures, manifest. The mortality rate among cases with neurological symptoms can be as high as 35% in children and 10% in adults, with long-term neurological deficits often being seen in survivors (2). In 1995, an outbreak of VEEV in Colombia and Venezuela resulted in over 100,000 human cases (3). In addition to natural outbreaks, VEEV is also a concern from a bioterrorism perspective, as it can be grown to high titers, requires a low infectious dose, and contains multiple serotypes. Both the former Soviet Union and the United States previously weaponized the virus, producing large quantities for their now defunct offensive bioweapons programs (4). Currently, vaccine strain TC83 is used in horses and for high-risk personnel; however, due to the low rate of seroconversion achieved with this vaccine (5) and its reliance on two single attenuating mutations (6), it is considered unfit for mass distribution (7). To date there are no FDA-approved therapeutics for VEEV infection, and further studies are required for clarification of the mechanisms associated with the underlying pathogenesis of VEEV.

Viral and host transcriptomic studies can provide a wealth of information on the underlying pathogenic mechanisms and interactions following the course of an infection. The use of high-throughput next-generation sequencing has led to the discovery of previously uncharacterized viruses and the establishment of numerous novel experimental systems redefining virus-host interactions. To date a number of studies have examined the alterations in the host transcriptome following VEEV infection. A comparative microarray analysis between cells persistently infected with VEEV and cells able to clear VEEV resulted in the identification of PARP12L as an antiviral factor (8). A molecular comparison utilizing microarrays of host-based responses to the TC83 strain was able to identify biomarkers differentiating between vaccine responder and vaccine nonresponder groups, as well as the involvement of interferon (IFN), interferon-induced pathways, Toll-like receptor (TLR), and interleukin 12 (IL-12)-related pathways (9). A study examining the role of adhesion and inflammatory factors in VEEV-infected CD-1 mice found viral modulation of the expression of extracellular matrix and adhesion genes, such as integrins (ItgαX, Itg2, 3, and 7), cadherins 1 and 2, vascular cell adhesion molecule 1, and intracellular adhesion molecule 1 (ICAM-1), in the brains of VEEV-infected mice (10). Follow-up experiments utilizing ICAM-1-knockout mice demonstrated reduced inflammation in the brain and a subsequent delay in the onset of neurological sequelae (10). A study by Sharma et al. utilized microarrays to analyze gene expression changes in the brain tissue of VEEV-infected mice over the course of an infection, discovering numerous immune pathways involved in antigen presentation, inflammation, apoptosis, and the traditional antiviral response (Cxcl10, CxCl11, Ccl5, Ifr7, Ifi27, Oas1b, Fcerg1, Mif, clusterin, and major histocompatibility complex [MHC] class II) (11). A second study by the same group identified the regulation of microRNAs (miRNAs) in the brains of VEEV-infected mice, which enabled the correlation of the miRNA changes with earlier mRNA expression data (11, 12). These analyses suggest that VEEV may be utilizing cellular miRNAs in order to regulate downstream mRNA, which may correspond with the VEEV-induced histological changes to the nervous system (11, 12).

In the current study, next-generation RNA sequencing (RNA-Seq) was used to identify clinically relevant alterations in the mRNA transcriptome of human astrocytes infected with wild-type (WT) VEEV strain Trinidad donkey (TrD). The analysis of host mRNAs by RNA-Seq provides novel insight into how a host responds to a viral infection through the identification of a wide and dynamic range of transcripts in an unbiased manner. Selective sequencing of mRNAs, specifically, polyadenylated [poly(A)] transcripts, which account for ∼1% of the entire transcriptome, enhances the detection of the most relevant and low-abundance transcripts (13). As VEEV has been shown to productively infect astrocytes both *in vitro* and *in vivo* (14, 15), we chose astrocytes as our model of interest. Astrocytes are the most abundant cell in the brain, outnumbering neurons by at least 5-fold (16), providing an abundant resource for viral replication within the brain. In addition to their well-described structural role in neuronal tissue, astrocytes play critical roles in other processes, including the regulation of blood flow and of the blood-brain barrier, synapse transmission, and the response to infection (16). VEEV-infected astrocytes have been shown to produce multiple cytokines, including IL-8, IL-17, interferon gamma (IFN-γ), and gamma interferon-induced protein 10, all of which were found to be associated with viral attenuation (14).

In order to obtain a dynamic view of the virus-host interactome, RNA-Seq was used to monitor changes in gene expression in VEEV TrD-infected astrocytes at 4, 8, and 16 h postinfection (hpi). By viewing the alterations at multiple early time points using triplicate biological replicates, a robust and dynamic range of information is generated, and this information provides an increase in both the power and the accuracy of detection of differentially expressed transcripts in a highly relevant clinical model (17). Among VEEV-infected cells, an increase in interferon-regulated genes, including IFIT1, IFIT2, IFIT3, and OASL, was observed. The increased expression of genes involved in the stress-induced unfolded protein response (UPR) pathway was also noted. Interestingly, VEEV infection resulted in an increase in early growth response protein 1 (EGR1), which may serve as a link between the two pathways. The identification of host mRNAs whose expression is altered following VEEV replication, specifically, EGR1 and its interactors up- and downstream, may provide novel host-based therapeutic targets critical for VEEV replication and a greater understanding of the underlying mechanisms underpinning alphavirus replication.

## MATERIALS AND METHODS

### Viral infections and plaque assays.

VEEV TrD was obtained from BEI Resources. All experiments with VEEV TrD were performed under biosafety level 3 (BSL-3) conditions. All work involving select agents is registered with the Centers for Disease Control and Prevention and was conducted at George Mason University's Biomedical Research Laboratory, which is registered in accordance with federal select agent regulations. For infections, VEEV was added to supplemented Dulbecco modified Eagle medium (DMEM) to achieve a multiplicity of infection (MOI) of 0.05, 0.5, or 5. Cells were infected for 1 h at 37°C and rotated every 15 min to ensure adequate coverage. The cells were then washed with phosphate-buffered saline (PBS), and complete growth medium was added back to the cells. Viral supernatants and cells were collected at various times postinfection for further analysis. Plaque assays were performed as previously described (18).

### mRNA isolation and poly(A) library preparation.

RNA from U87MG cells was purified from both VEEV TrD-infected (biosafety level 3) and mock-infected U87MG cells at 4, 8, and 16 hpi utilizing a mirVana isolation kit (Life Technologies). Quality control of purified RNA was then performed using an Agilent 2100 bioanalyzer, and an RNA integrity number (RIN) cutoff of 8 was utilized for all samples. An External RNA Controls Consortium (ERCC) RNA spike-in control mix was then added to the total RNA inputs (10 μg RNA) before poly(A) selection using a Life Technologies Dynabeads mRNA Direct kit. Preparation of a whole-transcriptome RNA library from purified mRNA was then performed using an Ion Total RNA-Seq kit (v2; Life Technologies). Quality control of the cDNA libraries was then performed using the Agilent 2100 bioanalyzer along with sterility testing for removal of libraries for sequencing from a BSL-3 to BSL-2 laboratory.

### RNA sequencing.

Library template preparation was performed on a One Touch 2 platform (Life Technologies). Next-generation RNA sequencing was performed on an Ion Torrent PGM platform and was carried out for each sample to assess the differential gene expression of infected versus uninfected cells over time.

### Data filtering and RNA-Seq analysis pipeline.

A total of ∼119 million sequencing reads and an average of 6.6 million reads per sample were used as the input into our analysis pipeline. Unless otherwise noted, downstream RNA-Seq analysis was carried out using the CLC bio Genomics Workbench (v7). Raw RNA-Seq reads were trimmed to remove any residual sequencing adapter fragments that remained on the 5′ or 3′ ends after sequencing. In addition, end trimming of reads was done using the modified Mott algorithm with a Q20 quality score, and any reads of less than 15 bp were discarded. Following read trimming, the reads were mapped to human genome hg19 with the following RNA-Seq parameters: a 10-hit limit for multiple mapped positions, a similarity fraction of 0.8, a length fraction of 0.8, a mismatch cost of 2, and an indel cost of 3. The expression level of individual genes and transcripts was calculated using the number of reads per kilobase of the exon model per million mapped reads (RPKM) method of Mortazavi et al. (19). In addition, unmapped reads were also mapped to the ERCC92 synthetic RNA sequence set (20), as well as to the VEEV reference genome (GenBank accession number L01442). In all samples, the correlation coefficient (*R*^2^) between the expected and the mapped number of reads for the ERCC92 spike-in controls was above 0.90. A summary of the overall sequencing results is shown in Table 1.

**TABLE 1:** Summary of RNA-Seq data^*a*^

Sample	No. of raw reads	No. of reads mapped to human genes	No. of genes	No. of viral reads detected	% of viral reads detected	No. of ERCC92 reads	% of ERCC92 reads
Mock-infected samples							
MOCK mRNA 4H R1	5,126,389	3,310,102	15,025		0.0	36,429	0.7
MOCK mRNA 4H R2	5,497,371	3,183,070	15,873		0.0	29,467	0.5
MOCK mRNA 4H R3ab	6,803,543	4,417,209	16,855	5	0.0	62,033	1.1
MOCK mRNA 8H R1ab	5,893,211	3,704,729	18,731	3	0.0	27,562	0.5
MOCK mRNA 8H R2	5,396,774	3,630,440	16,668	4	0.0	51,715	1.0
MOCK mRNA 8H R3ab	6,814,244	4,867,131	17,628	115	0.0	179,447	2.7
MOCK mRNA 16H R1	6,420,287	4,139,674	16,016	16	0.0	47,163	0.7
MOCK mRNA 16H R2	5,094,309	3,577,955	16,054	184	0.0	36,144	0.7
MOCK mRNA 16H R3	4,618,317	3,171,811	16,384	16	0.0	27,793	0.6
VEEV-infected samples							
VEEV mRNA 4H R1	4,925,927	2,827,089	15,561	58,659	1.2	31,132	0.6
VEEV mRNA 4H R2ab	6,283,014	2,976,033	14,015	55,558	0.9	53,668	0.9
VEEV mRNA 4H R3ab	9,019,118	5,870,150	17,349	116,978	1.3	107,303	1.2
VEEV mRNA 8H R1	5,310,960	2,322,137	15,701	999,715	19.0	20,211	0.4
VEEV mRNA 8H R2	5,549,103	2,679,642	15,806	1,044,308	19.0	25,541	0.5
VEEV mRNA 8H R3ab	8,334,045	4,731,269	18,355	1,738,006	21.3	45,409	0.6
VEEV mRNA 16H R1ab	10,549,349	2,740,917	14,769	4,725,888	45.1	28,680	0.3
VEEV mRNA 16H R2ab	8,470,485	2,296,478	13,732	4,228,398	50.1	26,992	0.3
VEEV mRNA 16H R3ab	9,690,240	2,353,572	14,540	3,548,202	36.8	24,144	0.3

a RNA was isolated from triplicate sets of mock- and VEEV-infected U87MG cell cultures, purified at 4, 8, and 16 hpi, and used to prepare cDNA libraries along with an ERCC92 synthetic RNA sequence set for downstream RNA-Seq (see Materials and Methods). A high-level summary of the overall RNA-Seq results is presented here (see Materials and Methods).

Postmapping filtering of all RNA-Seq data was carried out next to include only genes with at least one uniquely mapped read (26,230 genes remained across all data sets) and only those with a nonzero interquartile range across the entire experiment. Principal component analysis of the resulting filtered data set (13,906 genes in total) was carried out using raw counts of uniquely mapped reads (see Fig. 2A). The remaining RPKM expression values for each gene included in the filtered data set were subjected to quantile normalization with a 5% cutoff. A box plot of log_2_-transformed RPKM values for each sample before normalization is shown in Fig. 2B. The *R*^2^ value for pairwise sample-to-sample variation within each biological replicate set was observed to range from 0.89 to 0.99, indicating that our biological replicates were consistent and showed no strong bias (data not shown).

### Differential gene expression analysis.

Differentially expressed genes (DEGs) were identified using two approaches. First, the empirical analysis of differential gene expression algorithm, part of the edgeR Bioconductor package (21), was applied to the integrated data set of all 18 experiments using the default parameters and a false discovery rate-corrected *P* value. At each time point, infected and mock-infected samples were compared, and genes whose expression differed by more than 2-fold with a significance with a *P* value of ≤0.05 were provisionally considered to be differentially expressed.

In addition to the method described above, an orthogonal statistical test of differential expression was applied to the data using a statistical test developed by Baggerly et al. (22) to count the number of expressed sequence tags associated with individual genes, a common feature of both serial analysis of gene expression (SAGE) data and RNA-Seq data. When infected and mock-infected samples were compared, individual genes were provisionally considered differentially expressed when their expression differed by more than 2-fold with a significance with a *P* value of ≤0.05. Differentially expressed genes found to be in the intersection of the sets of genes identified by both of the methods outlined above were considered high-quality candidates and used as the starting point for further investigation.

### Clustering and GSEA.

Filtered, normalized expression data were subjected to k-means clustering using a Euclidian distance metric where genes were grouped by means of normalized gene expression (RPKM) values for each experimental condition. Clustering was fitted to 20 distinct clustering groups, and the individual gene expression profiles clustered were further tested for enrichment of gene ontology (GO) terms associated with individual genes. Gene annotations were obtained from Reactome, a database of biological pathway and gene functional annotations (23). Enrichment analysis was performed using two approaches. First, a hypergeometric test on GO annotations was carried out using an implementation of the GOStats package on each of the individual clusters obtained from k-means clustering (24). In addition, gene set enrichment analysis (GSEA) was carried out on the entire filtered data set using 100,000 permutations, while duplicates were removed and an analysis of variance was applied. A total of 1,419 categories passed a minimum feature size of 10 and were used for further investigation.

### Pathway analysis.

Cohorts of genes with shared patterns of expression over time were identified by k-means clustering. Those found to be enriched for DEGs were subsequently subjected to pathway analysis using the GeneMania system (25). Using an *ad hoc* manual approach, relevant pathways and the connections between them were identified on the basis of existing data in the literature coupled with the temporal gene expression data obtained from this study.

### qRT-PCR analysis.

Purified mRNA was converted to cDNA using a high-capacity RNA-to-cDNA kit (Life Technologies) according to the manufacturer's instructions. Analysis of the viral copy numbers was performed by quantitative reverse transcription-PCR (qRT-PCR) as previously described (26). Host expression of the following genes was assayed with TaqMan assays (indicated in parentheses): activating transcription factor 3 (ATF3; Hs00231069_m1), ATF4 (Hs00909569_g1), CEBPB (Hs00270923_s1), CEBPD (Hs00270931_s1), DDIT3 (Hs00358796_g1), FOS (Hs04194186_s1), JUN (Hs01103582_s1), EGR1 (Hs00152928_m1), IFI6 (Hs00242571_m1), IFIT1 (Hs01911452_s1), IFIT2 (Hs01922738_s1), IFIT3 (Hs01922738_s1), ISG15 (Hs01921425_s1), ISG20 (Hs00158122_m1), OASL (Hs00984387_m1), BIRC5 (Mm00599749_m1), and XIAP (Mm01311594_mH). Assays for 18S rRNA (Hs99999901_s1 or Mm04277571_s1) were used for normalization. Assays were performed according to the manufacturer's instructions using an ABI StepOne Plus instrument.

### Treatment with PERKi and collection for Western blot analysis.

U87MG cells were pretreated for 2 h with 10 μM the protein kinase RNA-like endoplasmic reticulum (ER) kinase (PERK) inhibitor (PERKi) GSK2606414 (catalog number 516535; EMD Millipore) or dimethyl sulfoxide (DMSO) in DMEM prior to infection with VEEV TrD (MOI, 5). After 1 h, the viral inoculum was removed and cells were washed with sterile PBS (1×). The medium was replaced with medium containing the inhibitor or DMSO. At 16 hpi, the medium was removed, and the cells were washed with PBS and then collected for Western blot analysis.

### Knockdown of EGR1 with siRNA.

U87MG cells seeded at 6.7 × 10^4^ cells per well in a 12-well plate were transfected with 50 nM siGenome SMARTpool EGR1 (catalog number M-006526-01; Dharmacon) or AllStar negative-control small interfering RNA (siRNA; catalog number 1027280; Qiagen), using 1.33 μl of the Dharmacon DharmaFECT 1 transfection reagent (catalog number T-2001-02). At 24 h posttransfection, cells were infected with VEEV TrD (MOI, 5) for 1 h. After infection the medium was replaced with fresh medium. At 25 h after infection, supernatants or cells were collected for analysis.

### Protein lysate preparation and Western blot analysis.

Protein lysate preparation and Western blot analysis were performed as previously described (27). Primary antibodies to the following were used: EGR1 (antibody 44D5; catalog number 4154; Cell Signaling), polyclonal anti-Venezuelan equine encephalitis virus TC83 (subtype IA/B) capsid protein (BEI Resources), CHOP (antibody L63F7; catalog number 2895; Cell Signaling), phosphorylated α subunit of eukaryotic initiation factor 2 (p-eIF2α; Ser51; antibody D9G8; catalog number 3398; Cell Signaling), ATF4 (antibody D4B8; catalog number 11815; Cell Signaling), activated caspase 3 (antibody Asp175; catalog number 9661; Cell Signaling), and horseradish peroxidase-conjugated β-actin (catalog number ab49900-100; Abcam).

### Immunofluorescence analysis.

U87MG cells were grown on coverslips in a 6-well plate, infected with VEEV TrD as described above, washed with PBS (without Ca and Mg), and then fixed with 4% formaldehyde. Cells were permeabilized with 0.5% Triton X-100 in PBS for 20 min and then washed twice with PBS. The cells were blocked for 10 min at room temperature in 3% bovine serum albumin in PBS. Primary antibodies consisting of a VEEV capsid protein (catalog number NR-9403; BEI Resources) diluted 1:600 and an EGR1 antibody (antibody 44D5; catalog number 4154; Cell Signaling) diluted 1:400 were incubated in fresh blocking buffer at 37°C for 1 h and washed 3 times for 3 min each time in 300 mM NaCl with 0.1% Triton X-100. Alexa Fluor 568 donkey anti-goat secondary antibody (catalog number A11057; Invitrogen) and Alexa Fluor 488 donkey anti-mouse secondary antibody (catalog number A21202; Invitrogen) diluted 1:400 were used as secondary antibodies and treated in the same manner as the primary antibodies. DAPI (4′,6-diamidino-2-phenylindole) diluted 1:1,000 was used to visualize the nuclei. Coverslips were mounted onto glass slides using 10 μl of Fluoromount G mounting medium (catalog number 0100-01; Southern Biotech). A Nikon Eclipse TE2000-U fluorescence microscope was used for fluorescence microscopy. Images were viewed using a 60× objective oil immersion lens. Five images of each sample were obtained, and a representative image of each sample is shown below. All images were subjected to four-line averaging. The images were processed through Nikon NIS-Elements AR Analysis (v3.2) software.

### CellTiter Glo and Caspase 3/7 Glo assays.

Wild-type and EGR1^−/−^ mouse embryonic fibroblasts (MEFs) were infected with TrD at various MOIs for an hour and then washed with PBS, and the medium was replaced. Cell viability was measured at 24 h postinfection using a Promega CellTiter luminescent cell viability assay (catalog number G7571) according to the manufacturer's protocol. Luminescence was read using a Beckman Coulter DTX 880 multimode detector with an integration time of 100 ms per well. Similarly, caspase activation in infected wild-type and EGR1^−/−^ MEFs was measured at 24 h postinfection using a Promega Caspase 3/7 Glo assay (catalog number G8090) according to the manufacturer's protocol. Luminescence was read using the DTX 880 multimode detector with an integration time of 100 ms per well.

### Nucleotide sequence accession numbers.

The raw sequencing data for all RNA-Seq runs included in this work are publically available in the NCBI BioProject database under accession number PRJNA300864 (http://www.ncbi.nlm.nih.gov/bioproject/PRJNA300864).

## RESULTS

### VEEV replication kinetics in U87MG astrocytes.

**FIG 1:**
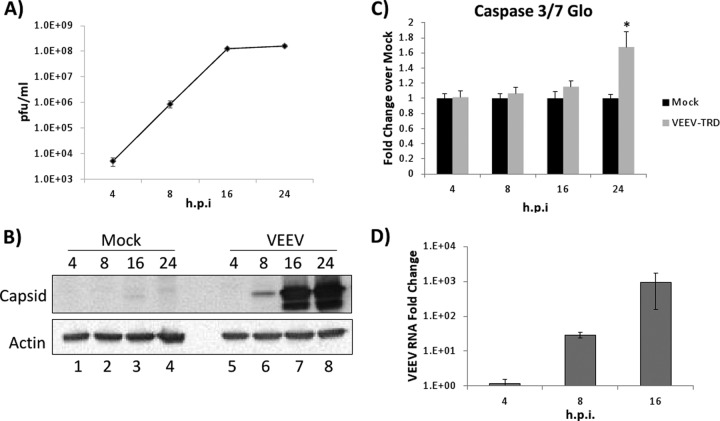
VEEV replication kinetics in U87MG cells. U87MG cells were infected with VEEV TrD (MOI, 5). (A) Viral supernatants were collected at 4, 8, 16, and 24 hpi, and viral titers were determined by plaque assays. (B) Protein lysates were collected at 4, 8, 16, and 24 hpi (as indicated above each lane), and Western blot analysis was performed with anticapsid and antiactin antibodies. (C) U87MG cells were mock treated or infected as described in the legend to panel A. Caspase 3/7 activity was analyzed using the Caspase 3/7 Glo assay (Promega). *, *P* < 0.01. (D) RNA was extracted using a miRVana kit, and VEEV RNA was quantitated by qRT-PCR. The data shown are the fold change in normalized gene expression (compared to that for the VEEV-infected sample at 4 hpi) determined by the ΔΔ*C_T_* threshold cycle (*C_T_*) method.

VEEV replicates *in vivo* in monocytes, macrophages, neurons, and astrocytes (14). Common cell lines used to study VEEV infection include Vero and BHK cells; in this study, U87MG astrocytes were chosen as an *in vitro* model due to their physiological relevance and greater clinical significance. Initial experiments were performed to characterize viral replication in U87MG cells. VEEV replication kinetics in U87MG cells were measured using plaque assays and by monitoring viral protein and RNA expression levels and the cytopathic effect (CPE) on the infected cells (Fig. 1). Viral release was observed as early as 4 hpi, with ∼4 log units of virus being observed, followed by a consistent increase in replication at 8 and 16 hpi (Fig. 1A). Viral replication peaked at 16 hpi, and no additional increase in viral titers was observed at 24 hpi. Viral capsid expression followed a similar pattern, with protein being detected at 8 hpi and expression plateauing at 16 hpi (Fig. 1B). Among infected U87MG cells, a significant CPE was observed by microscopy at 24 hpi, with little to no CPE being detected at 16 hpi (data not shown). Consistent with these observations, increased caspase 3/7 activity was observed only at 24 hpi (Fig. 1C). On the basis of these data, times of 4, 8, and 16 hpi, reflecting the early, middle, and late stages of the viral life cycle, respectively, were selected for RNA-Seq analysis in order to provide a dynamic view of the host-pathogen transcriptome profile.

### RNA sequencing analysis of VEEV-infected astrocytes.

mRNA from triplicate sets of mock- and VEEV-infected U87MG cell cultures was isolated, purified at 4, 8, and 16 hpi, and used to prepare cDNA libraries for downstream RNA-Seq (see Materials and Methods). A high-level summary of the RNA-Seq results is shown in Table 1. VEEV RNA samples were assayed by quantitative RT-PCR at each time point as a control to demonstrate the increasing viral RNA load over time (Fig. 1D), consistent with the increasing number of RNA-Seq reads mapped to the VEEV genome at later time points (Table 1).

**FIG 2:**
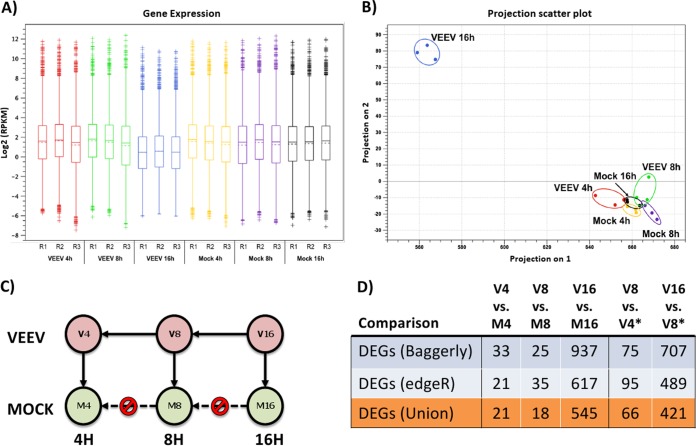
RNA-Seq data analysis. (A) Box plot of log_2_-transformed RPKM expression values from each individual biological replicate. (B) Principal component analysis of mock-infected versus VEEV-infected U87MG cells at 4, 8, and 16 hpi. (C) Diagram of the process used for the comparative analysis approach to identify DEGs at each sampling time point. (D) Total number of DEGs identified for each comparison shown in panel C using two different statistical methods (see Materials and Methods). The union of DEGs identified by both methods is shown in orange. *, the comparison includes those genes in VEEV-infected cells whose expression also did not change significantly in mock-infected cells at the same time points. V4, V8, and V16, VEEV-infected cells at 4, 8, and 16 hpi, respectively; M4, M8, and M16, mock-infected cells at 4, 8, and 16 hpi, respectively.

For RNA-Seq analysis, individual genes were expressed as the number of reads per kilobase of the exon model per million mapped reads (RPKM) (19). Log_2_-normalized RPKM expression values for each experimental sample are shown in Fig. 2A and can be found in Data Set S1 in the supplemental material. Minimal sample-to-sample variation in expression values within biological replicates was consistently detected (*R*^2^ > 0.89 for all replicates; data not shown). In addition, intersample variation was also found to be minimal when it was tested pairwise across the entire experiment by using RPKM values for ERCC97 synthetic spike-in control RNAs (*R*^2^ > 0.90 for all comparisons; data not shown).

**FIG 3:**
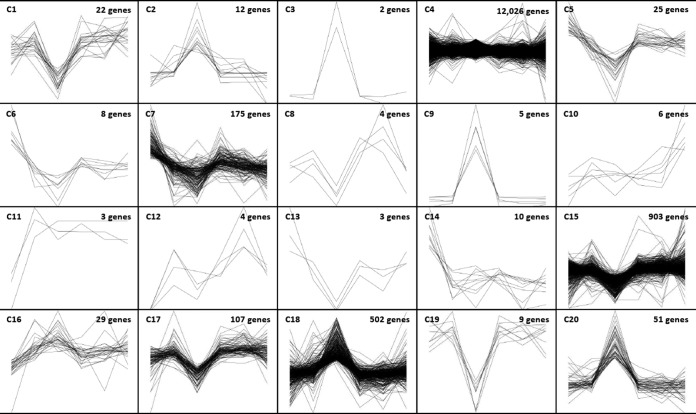
k-means clustering of expressed genes. k-means clustering (20 groups) of genes based on Euclidean distance metrics of expression over time for both mock- and VEEV-infected U87MG cells. Data were normalized on the basis of the group means and are plotted as the relative transformed expression level for each gene. Each cluster (C) has six data points per gene (VEEV-infected cells at 4, 8, and 16 hpi followed by mock-infected cells at 4, 8, and 16 hpi). See Data Set S2 in the supplemental material for a complete list of genes expressed within each cluster.

**FIG 4:**
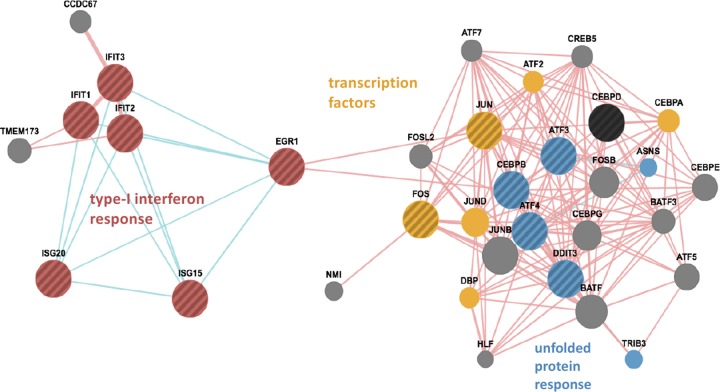
Network of type I interferon response- and UPR-related genes. Large circles, differentially expressed genes; small circles, genes with no significant change in expression; red circles, type I interferon response factors; yellow circles, genes regulating DNA transcription; blue circles, unfolded protein response genes; red lines, genes involved in physical protein-protein interactions; blue lines, genes involved in a common pathway. This network was seeded with k-means clusters 18 and 20, and many ribosomal protein genes were removed.

**FIG 5:**
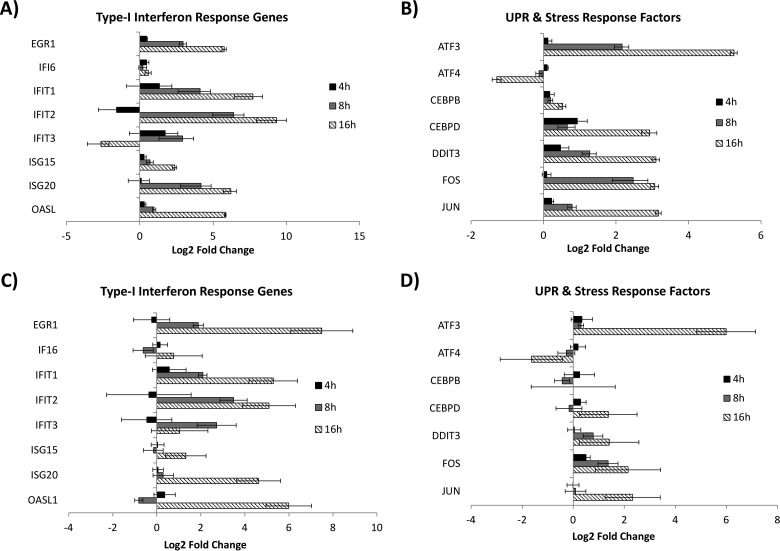
Next-generation RNA-Seq results and results of confirmatory qRT-PCR analysis. (A and B) RNA-Seq data for interferon response genes (A) and UPR and stress response genes (B). The data shown in panels A and B are log_2_-transformed values of the fold change of normalized gene expression (RPKM). Error bars are the ratiometric standard deviations of a two-component log function (VEEV-infected cells/mock-infected cells). (C and D) RNA was extracted using a miRVana kit, and gene expression was determined by qRT-PCR using TaqMan assays for interferon response genes (C) or for UPR and stress response genes (D). The data shown in panels C and D are log_2_-transformed values of the fold change of normalized gene expression determined by the ΔΔ*C_T_* threshold cycle (*C_T_*) method.

As anticipated, two-component principal component analysis of the RNA-Seq data for mock-infected cells versus VEEV-infected cells showed a clear separation of the samples at 16 hpi from the samples at earlier time points (Fig. 2B). However, the clustering of VEEV-infected samples with mock-infected samples at earlier time points suggested that the response to viral infection was limited to a narrow subset of early response genes, thus placing a higher burden of proof on identifying differentially expressed genes (DEGs) during the first few hours of infection. Along these lines, two orthogonal methods were used to identify DEGs suitable for further characterization: the edgeR method (21) and the method developed by Baggerly et al. (22). Genes identified by one method were provisionally considered DEGs, and those identified by both methods were candidate DEGs to be confirmed by qRT-PCR. In addition to comparing individual gene expression values for mock-infected cells and VEEV-infected cells at each time point, gene expression values were also compared serially within each time series of VEEV-infected cells for genes that did not show any statistically significant changes in expression in mock-infected cells. A schematic of the comparative analysis is shown in Fig. 2C. The number of statistically significant DEGs identified by each of these comparisons is shown in Fig. 2D. Furthermore, k-means clustering (against normalized RPKM values) was employed to identify gross changes in gene expression over time for cohorts of genes potentially sharing the same pathway or regulatory triggers (Fig. 3; see also Data Set S2 in the supplemental material). Gene set enrichment analysis (GSEA; see Material and Methods and Data Set S3 in the supplemental material) was carried out on each k-means cluster. In particular, cluster 20 (Table 2) was significantly enriched for genes involved in translational control, the type I interferon-mediated signaling pathway, and the unfolded protein response (UPR) pathway (GSEA *P* value < 0.01). Although there is a well-established connection between translational control and UPR, a novel connection between UPR and the type I interferon-mediated response in response to viral replication was suggested by pathway analysis (see Materials and Methods), implicating early growth response 1 (EGR1) as a potential bridge between these two pathways (Fig. 4). EGR1 belongs to cluster 20 and is strongly induced during VEEV infection, and several other genes associated with the interferon response belong to the same cluster: IRF1, IFIT1, IFIT2, ISG15, and ILF3. EGR1 has been associated with increases in the expression of activating transcription factor 3 (ATF3) (28), which is a key component of the UPR and which also belongs to cluster 20. This connection represented a potential bridge between the UPR pathway and the interferon response pathway, with EGR1 being one of the potential key transcription factors driving this connection. Consequently, 15 genes from this analysis were selected for further characterization by qRT-PCR (see below): ATF3, activating transcription factor 4 (ATF4), CEBPB, CEBPD, DDIT3/CHOP, EGR1, FOS, IFI6, IFIT1, IFIT2, IFIT3, ISG15, ISG20, JUN, and OASL. The expression values of these genes, as measured by RNA-Seq, are shown in Fig. 5A and B. Confirmatory qRT-PCR analysis indicated concordant gene expression (Fig. 5C and D). The interferon response genes induced are in agreement with those detected in previously published studies (11, 29, 30), and these genes served as an internal positive control. Moreover, the link between EGR1 and the interferon pathway has been demonstrated; EGR1 is induced by IFN-γ in mouse fibroblasts and by IFN-α, -β, and -γ in human fibroblasts (31, 32). EGR1 and the UPR pathway were selected for further analysis, as their role in VEEV infection has not been elucidated.

**TABLE 2:** k-means cluster 20 results

Reactome biological process annotation (accession no.)^*a*^	No of genes in^*b*^:		*P* value
	Full set	Subset	
Viral transcription (REACT_6152)	77	6	1.60E−06
Translational termination (REACT_1986)	80	6	2.00E−06
Viral reproduction (REACT_6145)	485	12	2.02E−06
Translational elongation (REACT_1477)	85	6	2.86E−06
SRP-dependent cotranslational protein targeting to membrane (REACT_115902)	102	6	8.29E−06
Viral infectious cycle (REACT_6167)	110	6	1.28E−05
Nucleus-transcribed mRNA catabolic process, nonsense-mediated decay (REACT_75886)	111	6	1.35E−05
Translational initiation (REACT_2159)	113	6	1.49E−05
Cellular protein metabolic process (REACT_17015)	446	10	3.90E−05
Type I interferon-mediated signaling pathway (REACT_25162)	45	4	5.96E−05
mRNA metabolic process (REACT_20605)	213	6	4.99E−04
Antigen processing and presentation of exogenous peptide antigen via MHC class I, TAP independent (REACT_111168)	9	2	7.90E−04
Translation (REACT_1014)	234	6	8.18E−04
RNA metabolic process (REACT_21257)	236	6	8.55E−04
Cytokine-mediated signaling pathway (REACT_25229)	171	5	1.29E−03
Platelet degranulation (REACT_318)	50	3	1.72E−03
Activation of signaling protein activity involved in unfolded protein response (REACT_18348)	62	3	3.19E−03
Regulation of immune response (REACT_11152)	36	2	0.01

a Biological process annotations obtained from Reactome for cluster 20. Reactome annotation identifiers are indicated for each annotation. Only traceable author submission (TAS)-classified annotations are considered. TAP, transporter associated with antigen processing; SRP, signal recognition particle.

b Full set, the total number of genes in the genome with an annotated biological process; subset, total number of differentially expressed genes with an annotated biological process.

### VEEV infection induces UPR late in infection.

**FIG 6:**
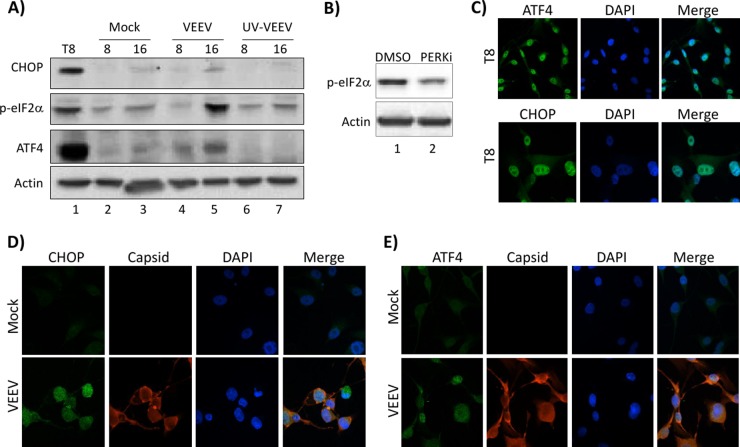
UPR is activated at later time points in VEEV infection. (A) U87MG cells were mock, VEEV, or UV-VEEV infected (MOI, 5), and protein lysates were collected at 4, 8, and 16 hpi (as indicated above each lane). Western blot analysis was performed using antibodies against CHOP, ATF4, p-eIF2α, and actin. T8, U87MG cells treated with tunicamycin for 8 h as a control for UPR induction. Results are representative of those from three independent experiments. (B) U87MG cells were pretreated with DMSO or a PERKi for 2 h prior to infection with VEEV TrD (MOI, 5) and replacement of the medium with drug-containing medium. Western blot analysis was performed using antibodies against p-eIF2α and actin. (C) U87MG cells were treated with tunicamycin for 8 h as a control for UPR induction. (D and E) U87MG cells were mock or VEEV infected (MOI, 5). At 16 h after infection, cells were fixed and probed with DAPI, anti-VEEV capsid, anti-ATF, or anti-CHOP primary antibodies and Alexa Fluor 488- and Alexa Fluor 568-labeled secondary antibodies. Slides were imaged on a Nikon Eclipse TE2000-U fluorescence microscope after immunofluorescence staining. Results are representative of those from two independent experiments.

The RNA-Seq and pathway analysis data indicated that UPR and stress response genes were induced after VEEV infection. During an infection, host cells respond to cellular stresses resulting from increased viral protein translation and secretion by triggering the onset of the UPR pathway. The UPR pathway is an adaptive cellular response activated by endoplasmic reticulum (ER) stress due to protein misfolding. In order to regulate cellular homeostasis during protein folding and secretion, the UPR pathway has developed three classes of sensors to ensure proper cellular regulation: inositol-requiring enzyme 1 (IRE1), protein kinase RNA-like ER kinase (PERK), and activating transcription factor 6 (ATF6) (33, 34). During VEEV infection, the PERK arm of the UPR appeared to be altered, as two critical regulators of this pathway were differentially expressed: ATF4 and CHOP (DDIT3) (35). To determine if DEGs altered subsequent protein expression, Western blot analysis was performed for CHOP, ATF4, and phosphorylated eIF2α (p-eIF2α). Tunicamycin, a glycosylation inhibitor and inducer of UPR (36), was included as a positive control. A time course analysis of U87MG cells treated with 1 μM tunicamycin indicated that 8 h of treatment provided the most robust induction of UPR proteins (data not shown). VEEV-infected but not mock-infected or UV-inactivated VEEV (UV-VEEV)-infected cells displayed a dramatic increase in p-eIF2α expression and a modest but consistent increase in CHOP and ATF4 expression at 16 hpi (Fig. 6A). No change in protein expression was observed at 4 hpi (data not shown). Confocal microscopy confirmed CHOP and ATF4 upregulation, demonstrating a more robust and nuclear staining pattern in VEEV-infected cells than in mock-infected cells (Fig. 6C to E). While ATF4 protein expression levels increased, ATF4 mRNA abundances decreased following VEEV infection (Fig. 5B and D). These results are consistent with the observation that ATF4 expression is regulated at the translational level upon UPR induction (37). As eIF2α can be phosphorylated by multiple kinases (PERK, protein kinase double-stranded RNA dependent [PKR], general control nonderepressible-2 [GCN2], and heme-regulated inhibitor [HRI]) (38), the PERK inhibitor (PERKi) GSK2606414 was used to determine if the observed phosphorylation was PERK dependent. Treatment of VEEV-infected cells with PERKi resulted in a marked decrease in eIF2α phosphorylation (Fig. 6B). These results indicate that PERK contributes to eIF2α phosphorylation but that there is likely an additional kinase contributing to the phosphorylation event. Collectively, these findings indicate that the PERK arm of the UPR pathway is induced at later time points following VEEV infection.

### EGR1 is upregulated in infected cells and localizes to the nucleus.

**FIG 7:**
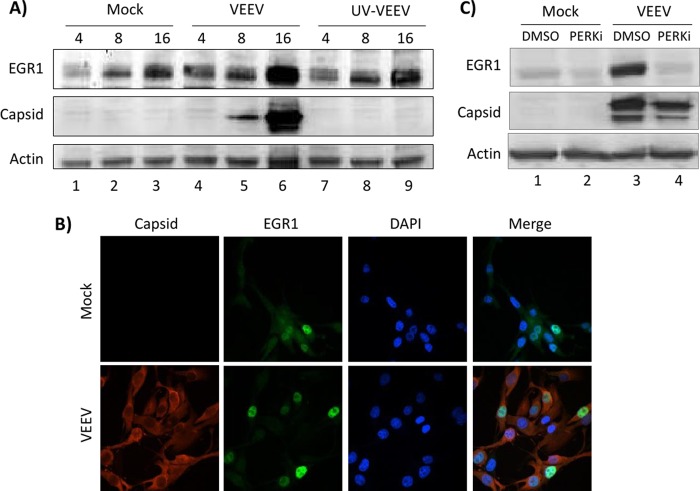
EGR1 is upregulated in infected cells and localizes to the nucleus. (A) U87MG cells were mock, VEEV, or UV-VEEV infected (MOI, 5), and protein lysates were collected at 4, 8, and 16 hpi (as indicated above each lane). Western blot analysis was performed using antibodies against EGR1, capsid, and actin. Results are representative of those from two independent experiments. (B) U87MG cells were mock or VEEV infected (MOI, 5). At 16 h after infection, cells were fixed and probed with DAPI, anti-VEEV capsid, and anti-EGR1 primary antibodies and Alexa Fluor 488- and Alexa Fluor 568-labeled secondary antibodies. Slides were imaged on a Nikon Eclipse TE2000-U fluorescence microscope after immunofluorescence staining. Results are representative of those from two independent experiments. (C) U87MG cells were pretreated with DMSO or a PERKi, infected with VEEV TrD (MOI, 5), and posttreated with drug-containing medium. Protein lysates were collected at 16 hpi. Western blot analysis was performed using antibodies against EGR1, capsid, and actin.

EGR1 is a transcription factor that can be induced by numerous signals, including oxidative stress, hypoxemia, and growth factors (39, 40). It can also be activated upon infection by both DNA and RNA viruses, including Epstein-Barr virus, mouse hepatitis virus, murine coronavirus, and Japanese encephalitis virus (41–43). Treatment of MEFs with the UPR activator thapsigargin has been shown to induce EGR1 expression in a PERK-dependent manner (44). Given the link between EGR1 and UPR and the robust induction of EGR1 mRNA expression following VEEV infection (Fig. 4 and 5), EGR1 was chosen for further study. EGR1 protein expression after VEEV infection was analyzed by Western blot analysis. As previous studies have indicated that EGR1 can be activated by mouse hepatitis virus independently of virus replication (likely due to cellular membrane disruption following entry) (41), a UV-inactivated virus control (UV-VEEV) was included. EGR1 protein levels were increased following VEEV infection compared to those in mock-infected cells and UV-VEEV-infected cells (Fig. 7A; compare lanes 3, 6, and 9). The most dramatic upregulation of EGR1 occurred at 16 hpi; this correlates with the highest levels of VEEV capsid production (Fig. 1B). Following induction, EGR1 has been shown to translocate to the nucleus to induce gene expression through binding to the Egr binding sequence (EBS) [GCG(G/T)GGCG] (40, 45). Confocal microcopy revealed high levels of EGR1 in the nuclei of infected cells, whereas only low levels of both nuclear and cytoplasmic EGR1 were detected in mock-infected cells (Fig. 7B). PERKi treatment of VEEV-infected cells resulted in a complete loss of EGR1 induction (Fig. 7C), indicating that EGR1 was induced in a PERK-dependent fashion. These results demonstrate that EGR1 protein levels and nuclear localization are increased following VEEV infection and that the induction of EGR1 is dependent on PERK.

### The loss of EGR1 inhibits VEEV-induced apoptosis but does not alter VEEV replication kinetics.

**FIG 8:**
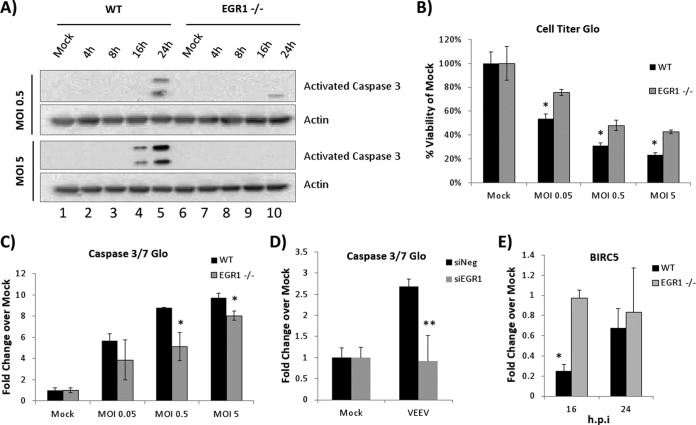
The loss of EGR1 reduces VEEV-induced apoptosis. (A) EGR1^−/−^ and WT MEFs were infected with VEEV at an MOI of 0.5 or 5. Protein lysates were prepared at 4, 8, 16, and 24 hpi and separated by SDS-PAGE, and Western blot analysis was performed using antibodies against cleaved caspase 3 and actin. Mock-infected cells were included as a control. Results are representative of those from two independent experiments. (B) EGR1^−/−^ and WT MEFs were infected with VEEV at an MOI of 0.05, 0.5, or 5. At 24 hpi cells were analyzed for viability using the CellTiter Glo assay (Promega). Mock-infected cells were included as a control, and their viability was set to 100%. *, *P* < 0.05 (comparison of WT and EGR1^−/−^ cells at the same MOI). Results are representative of those from two independent experiments. (C) EGR1^−/−^ and WT MEFs were infected with VEEV at an MOI of 0.05, 0.5, or 5. At 24 hpi caspase 3/7 activity was analyzed using the Caspase 3/7 Glo assay (Promega). Mock-infected cells were included as a control, and the fold change value for mock-infected cells was set to a value of 1. *, *P* < 0.05 (comparison of WT and EGR1^−/−^ cells at the same MOI). Results are representative of those from two independent experiments. (D) U87MG cells were transfected with either a negative-control siRNA (siNeg) or siRNA targeting EGR1 (siEGR1). At 48 h posttransfection, cells were infected with VEEV (MOI, 5). At 24 hpi caspase 3/7 activity was analyzed using the Caspase 3/7 Glo assay. The fold change values for mock-infected cells were set to a value of 1. **, *P* < 0.001. (E) EGR1^−/−^ and WT MEFs were mock or VEEV infected (MOI, 5). RNA was prepared, and gene expression was determined by qRT-PCR using a TaqMan assays for BIRC5 (survivin). The data shown are the values of the fold change of normalized gene expression determined by the ΔΔ*C_T_* threshold cycle (*C_T_*) method. *, *P* < 0.005 (comparison of VEEV-infected WT and EGR1^−/−^ cells).

As EGR1 influences cell survival and apoptosis (46), the impact of EGR1 on VEEV-induced cell death was assessed. Caspase 3 cleavage was observed in WT MEFs at 24 hpi when they were infected at an MOI of 0.5 and started as early as 16 hpi when they were infected at an MOI of 5 (Fig. 8A). In contrast, EGR1^−/−^ cells showed little to no detectable caspase cleavage following infection with VEEV. Two sets of experiments were performed to quantitatively confirm these results: CellTiter Glo assays to measure total cell viability (ATP production) and Caspase 3/7 Glo assays to measure caspase 3/7 activity. Both WT and EGR1^−/−^ MEFs displayed dose-dependent decreases in cell viability following VEEV infection, with EGR1^−/−^ cells having significantly more viable cells at each MOI examined (Fig. 8B). Concordantly, a dose-dependent increase in caspase 3/7 activity was observed following VEEV infection, with EGR1^−/−^ cells demonstrating reduced caspase 3 activity at MOIs of 0.5 and 5 (Fig. 8C). These results were replicated in U87MG cells transfected with siRNA targeting EGR1 (Fig. 8D).

EGR1 has been shown to negatively regulate the transcription of BIRC5 (survivin), an inhibitor of apoptosis (IAP) family member (47). RNA-Seq data indicated that BIRC5 gene expression was decreased following VEEV infection: log_2_-transformed fold change values of normalized gene expression were −1.16, −1.18, and −1.50 at 4, 8, and 16 hpi, respectively (see Table S1 in the supplemental material and NCBI BioProject accession number PRJNA300864). WT and EGR1^−/−^ MEFs were used to determine if EGR1 influenced BIRC5 gene expression following VEEV infection. BIRC5 expression was significantly decreased at 16 hpi in VEEV-infected WT MEFs, but this reduction was not observed in VEEV-infected EGR1^−/−^ MEFs (Fig. 8E). Expression of the gene for the X-linked inhibitor of apoptosis (XIAP), another IAP family member, was not significantly differentially altered after infection (data not shown). Collectively, these results demonstrate that EGR1 contributes to VEEV-induced apoptosis.

**FIG 9:**
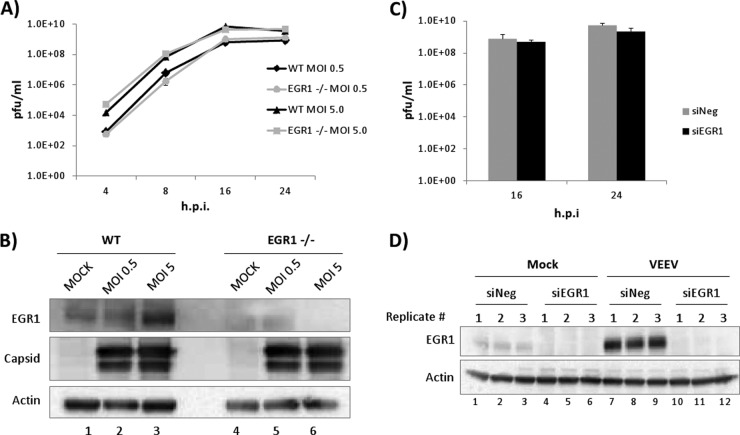
The loss of EGR1 does not alter VEEV replication kinetics. (A) EGR1^−/−^ and WT MEFs were infected with VEEV (MOI, 0.5 or 5.0). Viral supernatants were collected at 4, 16, and 24 hpi and analyzed by plaque assays. The average from biological triplicates is shown. (B) Protein lysates from EGR1^−/−^ and WT MEFs were separated by SDS-PAGE, and Western blot analysis was performed using antibodies against EGR1, capsid, and actin. (C) U87MG cells were transfected with a negative-control siRNA (siNeg) or siRNA targeting EGR1 (siEGR1). At 48 h posttransfection, cells were infected with VEEV (MOI, 5) or mock infected. Viral supernatants were collected at 16 and 24 hpi, and plaque assays were performed. The average from biological triplicates is shown. (D) Cells were treated as described in the legend to panel C. At 24 h postinfection, cells were collected for Western blot analysis. The results for three independent biological replicates are shown.

VEEV replication kinetics were determined for both EGR1^−/−^ and WT MEFs to determine the relevance of EGR1 in viral replication. Cells were infected at two different MOIs (0.5 and 5), and viral supernatants were collected at 4, 8, 16, and 24 hpi and analyzed by plaque assay. The replication kinetics were similar between EGR1^−/−^ and WT MEFs at both MOIs, with titers peaking at 16 hpi (Fig. 9A). A lack of EGR1 expression was confirmed by Western blotting (Fig. 9B). These results were replicated in U87MG cells transfected with siRNA targeting EGR1. Transfection of siRNA targeting EGR1 resulted in a >90% decrease in EGR1 protein expression (Fig. 9D) without any significant effect on viral replication (Fig. 9C). These results suggest that the decrease in apoptosis observed in EGR1^−/−^ MEFs was not due to altered VEEV replication kinetics.

## DISCUSSION

Despite being recognized as an emerging threat, relatively little is known about the virulence mechanisms of alphaviruses, largely due to a knowledge gap in the host-pathogen interactome. VEEV infection often results in fatal encephalitis and is known to inhibit both cellular transcription and translation in order to downregulate the innate immune response (1, 48). In contrast, in the CNS VEEV has been shown to upregulate numerous genes in both the inflammatory response and apoptotic pathways (1, 48). Specifically, numerous proinflammatory cytokines, including interleukin-1β (IL-1β), IL-6, IL-12, glycogen synthase kinase 3β, inducible nitric oxide synthase, and tumor necrosis factor alpha (TNF-α), have all been shown to play a role in VEEV pathogenesis (49–53). The use of high-throughput next-generation sequencing technologies, such as RNA-Seq, allows an in-depth and unbiased look into the virus-host transcriptome, thus enabling changes in the expression of specific mRNAs to be connected with phenotypic outcomes. To this end, identification of critical differentially expressed transcripts among clinically relevant infected cells will help lead to a greater understanding of viral pathogenesis and may prove beneficial for the identification of therapeutic targets.

In this study, network analysis/RNA-Seq data and the results of protein expression studies revealed that VEEV infection resulted in activation of the PERK arm of the UPR pathway, including the activation of ATF4, CHOP, and eIF2α phosphorylation. Several alphaviruses have previously been reported to hijack key components of the UPR pathway in order to promote viral replication, as the reliance of enveloped viruses on the ER for the synthesis of viral envelope-associated glycoproteins and their transport to the plasma membrane often stresses the ER due to rapid viral protein production (54, 55). Modulation of the UPR is not unique to alphaviruses; rather, it is a shared trait of many positive-sense RNA viruses. Dengue virus has been shown to suppress PERK by inhibiting continued eIF2α phosphorylation in order to inhibit immediate apoptosis, increasing viral protein translation and extending the length of productive viral replication (34). Studies with hepatitis E virus (HEV) have demonstrated that expression of HEV capsid protein open reading frame 2 (ORF2) activates the expression of CHOP and ATF4 (56). In HEV, ORF2 was shown to stimulate CHOP through both ER stressors and amino acid response elements (AARE) through interaction with ATF4 (56).

The results shown here indicate that during VEEV infection, initiation of the UPR pathway and subsequent activation of EGR1 play a role in the outcome of virus-induced apoptosis. During the initial detection of ER stress, PERK is able to identify misfolded proteins in the lumen of the ER and phosphorylates eIF2α in order to initiate prosurvival pathways in the UPR through the general inhibition of protein synthesis (33, 34). VEEV appears to induce the UPR and promote increased eIF2α phosphorylation, which results in the translational inhibition of most mRNAs, while UPR selectively increases the translation of ATF4. ATF4 is responsible for the expression of genes that encode proteins involved in apoptosis, redox processes, amino acid metabolism, and ER chaperone recruitment and is a well-known mediator of the PERK pathway and CHOP (33, 34). CHOP activation facilitates the increased expression of cellular chaperones in order to counteract the buildup of misfolded proteins (57). Failure to suppress protein misfolding in persistently stressed cells, such as during a viral infection, can then result in activation of the proapoptotic transcription factor CHOP, leading to suppression of the antiapoptotic protein B cell lymphoma-2 (Bcl-2). CHOP can also function as a prosurvival transcription factor by dephosphorylating eIF2α through activation of the DNA damage-inducible protein (GADD34) in a self-regulating feedback look (33, 34). However, the data presented here support a model whereby VEEV infection leads CHOP to function in its proapoptotic role, as no change in GADD34 gene expression was detected by RNA-Seq analysis.

While the UPR was induced following VEEV infection, robust activation was not observed until later time points after infection. This is somewhat surprising, as VEEV infection is expected to induce significant ER stress due to the massive production of viral proteins during the course of an acute robust infection. The structural proteins of VEEV are translated from the viral subgenomic RNA into polyproteins on the rough ER. The E1 and pE2 precursor glycoproteins are then assembled as heterodimers in the ER, undergoing conformational changes requiring numerous chaperones (1, 58). It is possible that VEEV has developed mechanisms to subvert the induction of the UPR. In order to counteract the UPR, the nonstructural proteins (nsPs) of Chikungunya virus (CHIKV) have been shown to inhibit expression of ATF4 and other known UPR target genes, including GRP78/BiP, GRP94, and CHOP (59). Through nsP activity, CHIKV has developed a means of suppressing the UPR activity resulting from viral glycoprotein-induced ER stress, thus preventing immediate autophagy and apoptotic activation. The VEEV capsid is responsible for interfering with nucleocytoplasmic trafficking and inhibiting rRNA and mRNA transcription and has been implicated in the regulation of type I IFN signaling and the antiviral response through the regulation of both viral RNA and protein production (1, 48, 60). Therefore, we hypothesize that the ability of the VEEV capsid to inhibit cellular transcription and block nucleocytoplasmic trafficking results in delayed induction of the UPR.

The results of a detailed network analysis based on existing data in the literature, coupled with the temporal gene expression profiles obtained from this study, point toward EGR1 being an important node in the novel link between VEEV activation of the type I interferon response and UPR. EGR1 is known to form a DNA binding complex with C/EBPB, a critical dimerization partner of CHOP (61). Previous studies have demonstrated that the nuclear localization of CHOP may act as an inducer of EGR1 and that CHOP may act as a transcriptional cofactor for regulation of C/EBPB-EGR1 target genes (61). The results of the Western blot and microscopy analysis presented in this study support this model, as VEEV infection was found to increase both the overall levels and the nuclear distribution of CHOP along with those of EGR1. Previous studies demonstrated EGR1 mRNA induction by IFN-γ in mouse fibroblasts and by TNF-α, TNF-β, IL-1, IFN-α, IFN-β, and IFN-γ in human fibroblasts (31, 32). EGR1, also known as Zif268 and NGF1-A, is a zinc finger protein and mammalian transcription factor. It has been implicated in cellular proliferation and differentiation, but it may also have proapoptotic functions, depending on the cell type and stimulus (62). Of particular interest, EGR1 directly controls proliferation when activated by the mitogen-activated protein kinase/extracellular signal-regulated kinase pathway in mitogen-stimulated astrocytes (63). Virus-induced changes in EGR1 expression have been observed in several *in vitro* systems. In HIV-1-infected astrocytes, EGR1 upregulation was found to be induced by Tat through transactivation of the EGR1 promoter, leading to cellular dysfunction and Tat-induced neurotoxicity (64). Increased amounts of EGR1 mRNA have also been demonstrated to act in a region-specific manner, corresponding temporally with viral RNA production in the brain tissues of rats infected with either rabies virus or Borna disease virus (65).

In summary, the current study demonstrates a potential link between UPR activation and EGR1. EGR1^−/−^ MEFs demonstrated lower levels of susceptibility to VEEV-induced cell death than wild-type MEFs, indicating that EGR1 modulates proapoptotic pathways following infection. Studies are under way to determine if alteration of the UPR through small molecule inhibitors or siRNA interference influences VEEV replication and/or cell death. To date the mechanisms underlying VEEV pathogenesis and subsequent neuronal degeneration have been only partially elucidated. Therefore, determining the role of EGR1 and UPR may play a significant role in the development of a novel therapeutic target resulting in decreased neuronal death and the subsequent neuronal sequelae that result from infection.

## Supplementary Material

Supplemental material
