# Supplementary material for: Venezuelan Equine Encephalitis Virus Induces Apoptosis through the Unfolded Protein Response Activation of EGR1
Source: J Virol. 2016 Mar 11;90(7):3558–72. doi: 10.1128/JVI.02827-15 (PMC4794670; doi:10.1128/JVI.02827-15)
Supplement: Supplemental material [file supp_90_7_3558__index.html]

Supplemental material 

# Venezuelan Equine Encephalitis Virus Induces Apoptosis through the Unfolded Protein Response Activation of EGR1

## Supplemental material

- Supplemental file 1 -

  Data Set S1 (Normalized mean RPKM.)

  XLSX, 6.8M
- Supplemental file 2 -

  Data Set S2 (Clusters.)

  XLSX, 216K
- Supplemental file 3 -

  Data Set S3 (GO annotations.)

  XLSX, 3.2M
